# First report of *Trichopodapictipennis* (Diptera, Tachinidae) for the Canary Islands

**DOI:** 10.3897/BDJ.12.e137821

**Published:** 2025-02-05

**Authors:** Pablo Caloca, Daniel Suárez, Gustavo Peña, Carlos Ruiz

**Affiliations:** 1 Departamento de Biología Animal, Edafología y Geología, Facultad de Ciencias, Universidad de La Laguna, San Cristóbal de La Laguna, Spain Departamento de Biología Animal, Edafología y Geología, Facultad de Ciencias, Universidad de La Laguna San Cristóbal de La Laguna Spain

**Keywords:** new records, parasitoid, faunistics, pest control, Macaronesia, introduced species, Phasiinae, Pentatomidae, *Nezaraviridula*

## Abstract

**Background:**

The genus *Trichopoda* Berthold, 1827 is distributed in the Neotropical and Nearctic Regions and some species are very important for biological control. During the last decades, the species *Trichopodapictipennis* Bigot, 1876 has received much attention. It is of Neotropical origin, but it has been introduced throughout the western Palaearctic, probably through exchanges that transported its main host, the 'southern green stink bug' *Nezaraviridula*.

**New information:**

*Trichopodapictipennis* is reported for the first time from the Canary Islands. To date, this introduced species has been detected throughout the island of Tenerife and in a single locality on the island of La Gomera. This finding confirms that this species of Neotropical origin is expanding its range across the Palaearctic realm of this species of Neotropical origin. Parasitised *Nezaraviridula* were collected and reared under laboratory conditions to document the complete life cycle of *T.pictipennis*. Its potential effects on its main host, as well as on the Canary fauna, are discussed.

## Introduction

The family Tachinidae (Calyptratae, Oestroidea) comprises ca. 8,500 valid species worldwide (O'Hara et al. 2020). This family is especially interesting, as all species are endoparasitoids of arthropods (Stireman et al. 2006) and several species visit flowers for feeding (Tooker et al. 2006). The genus *Trichopoda* Berthold, 1827, which includes the species commonly called ‘feather-legged flies’, is distributed in the Neotropical and Nearctic realms and some species are very important for biological control (Dios and Nihei 2020). During the last decades, the species *Trichopodapictipennis* Bigot, 1876 has received much attention. It is of Neotropical origin (Dios et al. 2021), but was unintentionally introduced in Italy on the 1980s, probably through exchanges that transported its only known main host, the ‘southern green stink bug' *Nezaraviridula* (Linnaeus, 1758) (Colazza et al. 1996). Since then, it has been spreading throughout the western Palaearctic. The second record of the species was in north-eastern Spain in 1995 (Peris 1998). During the last two decades, it has been detected in the following countries: France (Tschorsnig et al. 2000), Slovenia (de Groot et al. 2007), The Netherlands (Zeegers 2010), Italy (Cargnus et al. 2011), Israel (Freidberg et al. 2011), Croatia (Bystrowski 2012), Switzerland (Obrecht 2015), Portugal (Almeida et al. 2017), Malta (Darmanin and Cerretti 2019), Egypt (El-Hawagry et al. 2020), Albania, Cyprus, Greece, Turkey, Russia (Kazilas et al. 2020), Algeria (Meriem et al. 2021), Germany (Dios et al. 2021) and Morocco (Kettani et al. 2022). It is still unknown whether this expansion is the result of long-distance dispersal from Italy or if multiple introduction events occurred (Bystrowski 2012). A distributional study of *T.pictipennis* in Spain and Portugal, which revealed a strong affinity for coastal Mediterranean areas, suggested that the main way of entrance for this species might be port areas, probably associated with parasitised *N.viridula* individuals on fresh fruit and vegetables (Ricarte et al. 2020).

The Canary Islands, an oceanic archipelago within the Macaronesian Region, stands out for its high endemicity rate in arthropods (Oromí et al. 2015, Florencio et al. 2021). Diptera are relevant, as they constitute the second most species-rich insect order of the archipelago, with ca. 1,200 species, almost a third of them being endemics (Gobierno de Canarias 2024). In the Canary Islands, the tachinid assemblage has not received much attention historically, with only few contributions focusing specifically on this family (Báez et al. 1986, Tschorsnig et al. 2007, Suárez et al. 2020). In the present paper, *Trichopodapictipennis* is reported for the first time from the Canary Islands, more specifically from the islands of Tenerife and La Gomera.

## Materials and methods

The collected individuals were pinned and examined under a Zeiss Stemi 2000 stereomicroscope and individuals were identified to species level by using the key of Dios and Nihei (2020). For study of the female genitalia, the last abdominal segments of one specimen were dissected and placed in 10% potassium hydroxide (KOH). Once the genitalia were visible, they were placed in glycerine and examined under a stereomicroscope. Illustrations of the terminalia were made using the vector graphics editor Inkscape, based on photos taken with a Canon EOS 750D camera. The material is deposited in the collection of the Department of Animal Biology of the University of La Laguna, Tenerife (DZUL). Additionally, in order to document the complete life cycle of *T.pictipennis*, a sampling of its main host *Nezaraviridula* was conducted. Live specimens that had eggs on the integument, indicating that they were potentially parasitised, were collected and reared in the laboratory.

## Taxon treatments

### Trichopoda pictipennis

Bigot, 1876

5169D217-A6C8-5756-B1A3-F61E506F7612

#### Materials

- **Type status:** Other material. **Occurrence:** recordedBy: Gustavo Peña; individualCount: 1; lifeStage: adult; occurrenceID: CB27062D-1AF5-59F2-955F-4A40C2482BA8; **Taxon:** taxonID: https://www.gbif.org/es/species/5059806; scientificName: Trichopodapictipennis Bigot, 1876; order: Diptera; family: Tachinidae; genus: Trichopoda; **Location:** island: Tenerife; country: Spain; stateProvince: Santa Cruz de Tenerife; municipality: Fasnia; locality: Fasnia; decimalLatitude: 28.245555; decimalLongitude: -16.435555; georeferenceProtocol: GPS; **Identification:** identifiedBy: Gustavo Peña; dateIdentified: 2023; **Event:** eventDate: 19/01/2022; eventRemarks: On flowers of Argyranthemum frutescens (Asteraceae); **Record Level:** basisOfRecord: HumanObservation
- **Type status:** Other material. **Occurrence:** recordedBy: Gustavo Peña; individualCount: 1; lifeStage: adult; occurrenceID: D28506C1-567C-5AAA-B53A-D442F905DC51; **Taxon:** taxonID: https://www.gbif.org/es/species/5059806; scientificName: Trichopodapictipennis Bigot, 1876; order: Diptera; family: Tachinidae; genus: Trichopoda; **Location:** island: Tenerife; country: Spain; stateProvince: Santa Cruz de Tenerife; municipality: Fasnia; locality: Fasnia; decimalLatitude: 28.245555; decimalLongitude: -16.435555; georeferenceProtocol: GPS; **Identification:** identifiedBy: Gustavo Peña; dateIdentified: 2023; **Event:** eventDate: 20/06/2022; eventRemarks: On flowers of Zygophyllum fontanesii (Zygophyllaceae); **Record Level:** basisOfRecord: HumanObservation
- **Type status:** Other material. **Occurrence:** catalogNumber: DZUL-36962; recordedBy: Pablo Caloca; individualCount: 1; sex: female; lifeStage: adult; occurrenceID: CA2F2897-64C6-5DB7-BE32-193571A1A877; **Taxon:** taxonID: https://www.gbif.org/es/species/5059806; scientificName: Trichopodapictipennis Bigot, 1876; order: Diptera; family: Tachinidae; genus: Trichopoda; **Location:** island: Tenerife; country: Spain; stateProvince: Santa Cruz de Tenerife; municipality: Puerto de la Cruz; locality: Puerto de la Cruz; decimalLatitude: 28.402587; decimalLongitude: -16.556857; georeferenceProtocol: GPS; **Identification:** identifiedBy: Daniel Suárez; dateIdentified: 2023; **Event:** eventDate: 01/03/2022; eventRemarks: On leaves of Boseayervamora (Amaranthaceae); **Record Level:** basisOfRecord: PreservedSpecimen
- **Type status:** Other material. **Occurrence:** catalogNumber: DZUL-36963; recordedBy: Pablo Caloca; individualCount: 1; sex: male; lifeStage: adult; occurrenceID: 08A9DD48-C7BB-564B-A415-D59575239239; **Taxon:** taxonID: https://www.gbif.org/es/species/5059806; scientificName: Trichopodapictipennis Bigot, 1876; order: Diptera; family: Tachinidae; genus: Trichopoda; **Location:** island: Tenerife; country: Spain; stateProvince: Santa Cruz de Tenerife; municipality: Puerto de la Cruz; locality: Puerto de la Cruz; decimalLatitude: 28.402587; decimalLongitude: -16.556857; georeferenceProtocol: GPS; **Identification:** identifiedBy: Daniel Suárez; dateIdentified: 2023; **Event:** eventDate: 01/03/2022; eventRemarks: On leaves of Boseayervamora (Amaranthaceae); **Record Level:** basisOfRecord: PreservedSpecimen
- **Type status:** Other material. **Occurrence:** recordedBy: María de Fuentes; individualCount: 1; sex: male; lifeStage: adult; occurrenceID: ED223ECF-8281-5D7C-BAC5-01F6BB577CC3; **Taxon:** taxonID: https://www.gbif.org/es/species/5059806; scientificName: Trichopodapictipennis Bigot, 1876; order: Diptera; family: Tachinidae; genus: Trichopoda; **Location:** island: Tenerife; country: Spain; stateProvince: Santa Cruz de Tenerife; municipality: San Cristóbal de La Laguna; locality: San Cristóbal de La Laguna; decimalLatitude: 28.403688; decimalLongitude: -16.335111; georeferenceProtocol: GPS; **Identification:** identifiedBy: Pablo Caloca; dateIdentified: 2023; **Event:** eventDate: 28/02/2022; **Record Level:** basisOfRecord: HumanObservation
- **Type status:** Other material. **Occurrence:** catalogNumber: DZUL-36964; recordedBy: Pablo Caloca; individualCount: 1; sex: male; lifeStage: adult; occurrenceID: DEEF9D8F-D991-55FF-87CB-07D8A95011E0; **Taxon:** taxonID: https://www.gbif.org/es/species/5059806; scientificName: Trichopodapictipennis Bigot, 1876; order: Diptera; family: Tachinidae; genus: Trichopoda; **Location:** island: Tenerife; country: Spain; stateProvince: Santa Cruz de Tenerife; municipality: Los Realejos; locality: Los Realejos; decimalLatitude: 28.403688; decimalLongitude: -16.575571; georeferenceProtocol: GPS; **Identification:** identifiedBy: Pablo Caloca; dateIdentified: 2023; **Event:** eventDate: 16/01/2023; eventRemarks: On leaves of Boseayervamora (Amaranthaceae); **Record Level:** basisOfRecord: PreservedSpecimen
- **Type status:** Other material. **Occurrence:** recordedBy: Carlos Ruiz; individualCount: 1; sex: male; lifeStage: adult; occurrenceID: 226F34DD-88B0-5189-AD16-9EB4D917469D; **Taxon:** taxonID: https://www.gbif.org/es/species/5059806; scientificName: Trichopodapictipennis Bigot, 1876; order: Diptera; family: Tachinidae; genus: Trichopoda; **Location:** island: Tenerife; country: Spain; stateProvince: Santa Cruz de Tenerife; municipality: Tegueste; locality: Tegueste; decimalLatitude: 28.544774; decimalLongitude: -16.318082; georeferenceProtocol: GPS; **Identification:** identifiedBy: Carlos Ruiz; dateIdentified: 2023; **Event:** eventDate: 16/01/2023; eventRemarks: On flowers of Foeniculum vulgare (Apiaceae); **Record Level:** basisOfRecord: HumanObservation
- **Type status:** Other material. **Occurrence:** recordedBy: Eduardo Jiménez; individualCount: 1; lifeStage: adult; occurrenceID: 8A6D3CD8-1C58-5568-9C19-6901CA0988B7; **Taxon:** taxonID: https://www.gbif.org/es/species/5059806; scientificName: Trichopodapictipennis Bigot, 1876; order: Diptera; family: Tachinidae; genus: Trichopoda; **Location:** island: Tenerife; country: Spain; stateProvince: Santa Cruz de Tenerife; municipality: San Cristóbal de La Laguna; locality: Punta del Hidalgo; decimalLatitude: 28.544774; decimalLongitude: -16.318082; georeferenceProtocol: GPS; **Identification:** identifiedBy: Eduardo Jiménez; dateIdentified: 2023; **Event:** eventDate: 02/02/2023; eventRemarks: On flowers of Crithmum maritimum (Apiaceae); **Record Level:** basisOfRecord: HumanObservation
- **Type status:** Other material. **Occurrence:** recordedBy: Carmen Delia González; individualCount: 1; lifeStage: adult; occurrenceID: 2FD96D2F-7FC1-528F-B196-8CFBF2B23001; **Taxon:** taxonID: https://www.gbif.org/es/species/5059806; scientificName: Trichopodapictipennis Bigot, 1876; order: Diptera; family: Tachinidae; genus: Trichopoda; **Location:** island: Tenerife; country: Spain; stateProvince: Santa Cruz de Tenerife; municipality: San Miguel de Abona; locality: San Miguel de Abona; decimalLatitude: 28.030278; decimalLongitude: -16.627952; georeferenceProtocol: GPS; **Identification:** identifiedBy: Carlos Ruiz; dateIdentified: 2023; **Event:** eventDate: 05/02/2023; **Record Level:** basisOfRecord: HumanObservation
- **Type status:** Other material. **Occurrence:** recordedBy: Uquén Fernández; individualCount: 2; lifeStage: adult; occurrenceID: 93AA6364-7D8B-5233-BD62-D09B0112CBAC; **Taxon:** taxonID: https://www.gbif.org/es/species/5059806; scientificName: Trichopodapictipennis Bigot, 1876; order: Diptera; family: Tachinidae; genus: Trichopoda; **Location:** island: La Gomera; country: Spain; stateProvince: Santa Cruz de Tenerife; municipality: San Sebastián; locality: San Sebastián; decimalLatitude: 28.092127; decimalLongitude: -17.114348; georeferenceProtocol: GPS; **Identification:** identifiedBy: Pablo Caloca; dateIdentified: 2023; **Event:** eventDate: 29/01/2023; **Record Level:** basisOfRecord: HumanObservation

#### Diagnosis

**Figure 1.**
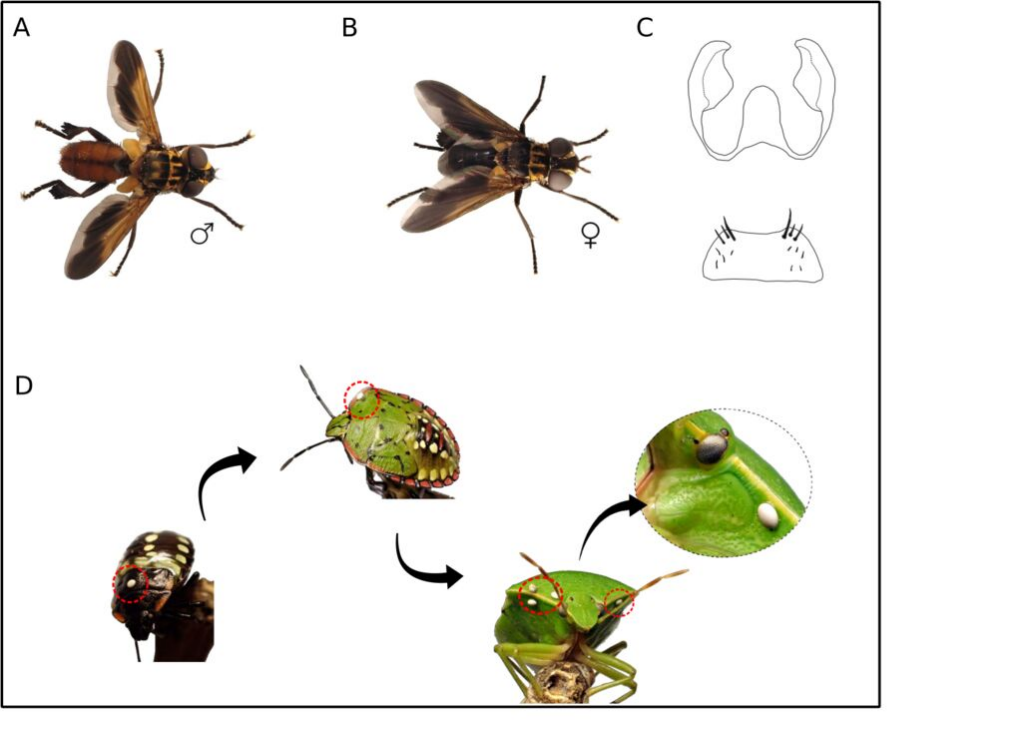
Habitus, genitalia and life cycle (partial) of *Trichopodapictipennis*. **A** Male of *Trichopodapictipennis*, photo by Pablo Caloca; **B** Female of *Trichopodapictipennis*, photo by Pablo Caloca; **C** Female genitalia of *Trichopodapictipennis*; **D** Parasitised individuals of *Nezaraviridula* at different life stages, photo by Pablo Caloca.

The collected individuals were identified as *Trichopodapictipennis* by the following combination of diagnostic characters. Males: yellow to orange abdomen, yellow scutellum, black wing with a yellow marking, lower calypter orange (Fig. 1A). Females: black abdomen and scutellum, black wing with a yellow marking (Fig. 1B). Female genitalia: sternite 7 as a trapezoidal plate, distal margin slightly rounded; sternite 8 lingulate, apically round; cercus subquadrate (Fig. 1C).

#### Biology

Parasitised individuals of *Nezaraviridula* were collected from leaves of *Boseayervamora* (Amaranthaceae) in the localities of Puerto de La Cruz and Los Realejos (north of Tenerife) on 17 December 2022 (Fig. 1D). *Trichopodapictipennis* full-grown larva emerged on 22 December 2022 and pupariated outside its host. The adult emerged on 8 January 2023.

## Discussion

**Figure 2.**
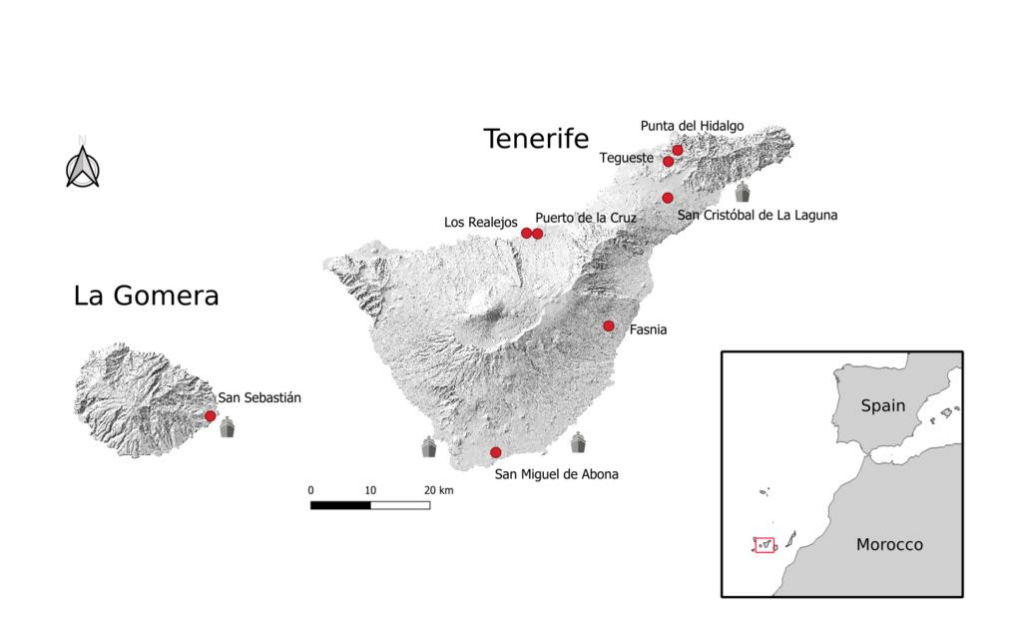
Map showing the localities where *T.pictipennis* has been reported (red dots). Commercial ports in Tenerife and La Gomera have been marked with a ship symbol.

The present record of *Trichopodapictipennis* is not only the first report of the species for the Canary Islands, but also for the Macaronesian Region (Arechavaleta et al. 2005, Borges et al. 2008, Borges et al. 2022, Gobierno de Canarias 2024). The species can be easily distinguished from any other tachinid species currently known from Macaronesia by the presence of feather-like setae on the hind tibia. *T.pictipennis* had previously been misidentified as *T.pennipes* Fabricius, 1781 throughout the Palaearctic realm until Dios et al. (2021). Its correct identification for further use in biological control is crucial to avoid harmful impacts on Pentatomidae native communities (Dios and Nihei (2020)). The distribution of *T.pictipennis* in the Canary Islands partially fits (a third of the records) with the commercial ports present at Tenerife and La Gomera (Fig. 2), possibly confirming the hypothesis of Ricarte et al. (2020) for mainland Spain. Two-thirds of the records were found far from commercial ports, possibly indicating expansions throughout the islands. This mode of entrance has also been proposed for other introduced species on the Canary Islands (Martínez and Barone 2006, López-dos-Santos et al. 2013, Ruiz et al. 2020, Lugo et al. 2022). However, this hypothesis should be tested with a phylogeographic study comprising material from across its distribution range, both native and introduced.

The main host of *T.pictipennis* is *Nezaraviridula* (Cerretti and Tschorsnig 2010, Tschorsnig 2017), which is present on all the major islands of the Canary Archipelago (Gobierno de Canarias 2024), implying the potential for *T.pictipennis* to spread to any island. This globally introduced species is known to feed on native plants in natural and agricultural ecosystems, thus causing economic damage to crops where it has been introduced, especially in subtropical and tropical regions (García et al. 1992, Esquivel et al. 2019). The recent arrival and establishment of *T.pictipennis* may have a regulatory impact on *N.viridula*. Although data collected in Italy suggests a moderate effect on parasitism rate (Salerno et al. 2002), differences in climatic conditions may lead to different rates on the Archipelago. Potentially, *T.pictipennis* could also shift to other Pentatomidae species. Outside its native area, there is only a single record of a host other than *N.viridula*, the Mediterranean species *Graphosomalineatum* (Linneaus, 1758) (Colazza et al. 1996, Cerretti and Tschorsnig 2010, Tschorsnig 2017). However, under laboratory conditions, *T.pictipennis* was able to parasitise additional three Australian native species belonging to different genera of Pentatomidae (Sands and Coombs 1999). The fact that there is just one known record of a host different from *N.viridula* suggests that T.pictipennis is a specialist. To date, we have only detected parasitism on *N.viridula*, with adults emerging from pupae 18 days after larvae emerged from its host. However, the idea of a potential niche shift should not be discarded. Insular biota exhibit several geographic, demographic and genetic characteristics that make them particularly vulnerable to invasive species (Leclerc et al. 2018, Russell and Kaiser-Bunbury 2019, Fernández-Palacios et al. 2021). In the Canary Islands, there are 38 native species of Pentatomidae, including seven endemic species. Therefore, it is imperative to study whether *T.pictipennis* can parasitise other species in the Canary Islands, with the aim of mitigating potential adverse impacts on the native Pentatomidae fauna.

## Supplementary Material

XML Treatment for Trichopoda pictipennis
